# Effect of Banana (*Musa* sp.) Peels Extract in Nanoemulsion Dosage Forms for the Improvement of Memory: *In Vitro* & *In Vivo* Studies

**DOI:** 10.2174/2211738510666220422135519

**Published:** 2022-11-14

**Authors:** Nur Achsan Al-Hakim, Irda Fidrianny, Kusnandar Anggadiredja, Rachmat Mauludin

**Affiliations:** 1 School of Pharmacy, Bandung Institute of Technology, Bandung, 40132, Indonesia

**Keywords:** Banana peels extract, nanoemulsion, antioxidant, acetylcholinesterase, tyrosinase, short-term memory, Y-maze, mice

## Abstract

**Background:**

Banana (*Musa* sp.) is a plant rich in phytochemical compounds, especially antioxidants, which are hypothesized to inhibit the activity of acetylcholinesterase, an enzyme associated with Alzheimer's Disease.

**Objective:**

This research aimed to study nanoemulsion preparations of Kepok banana (KEP-NE) and Tanduk banana (TAN-NE) peel extracts for their activities as antioxidants, acetylcholinesterase as well as tyrosinase inhibitors, and as agents to improve short-term memory.

**Methods:**

Nanoemulsion was prepared using a combination of high shear homogenization and ultrasonication. The antioxidant activity test was carried out using DPPH and ABTS methods. Meanwhile, memory improvement was studied in a mouse model with memory impairment induced by alloxan (120 mg/kg b.w) using the Y-maze apparatus. ELISA performed determination of acetylcholinesterase and tyrosinase inhibition.

**Results:**

Characterization of the nanoemulsion was performed to include particle size, antioxidant activity, acetylcholinesterase, and tyrosinase inhibition. The particle size and polydispersity index (PI) of KEP-NE and TAN-NE were 84.2 nm (PI: 0.280) and 94.1 nm (PI: 0.282), respectively. The antioxidant activity of DPPH showed that the respective IC_50_ values of KEP-NE and TAN-NE were 0.64 µg/mL and 1.97 µg/mL. At the same time, the values with the ABTS method were 1.10 µg/mL and 1.72 µg/mL, respectively. The IC_50_ of KEP-NE on acetylcholinesterase inhibition was 108.80 µg/mL, and that on tyrosinase inhibition was 251.47 µg/mL. The study of short-term memory in the Y-maze revealed that the groups Kepok peel extracts 100 and 300 mg/kg b.w and KEP-NE 100 and 300 mg/kg b.w significantly (*P* < 0.05) improved short-term memory.

**Conclusion:**

This study suggests that the nanoemulsion dosage form of Kepok banana peel extract has antioxidant and acetylcholinesterase inhibition and tyrosinase inhibition activities and could potentially be an adjunct alternative treatment for memory disorders. Modifying the smaller drug particle size contributes to the delivery system. The nanoemulsion can increase pharmacological activity.

## 1 INTRODUCTION

Memory disorder has been one of the burdens to healthcare, accounting for 65% of deaths worldwide [1]. This disorder covers dementia, Alzheimer’s, and amnesia [2]. An important contributing factor to memory loss is oxidative stress, where free radicals in the brain can damage hippocampal tissues [3]. Oxidative stress is characterized by-an imbalance between the production of Reactive Oxygen Species (ROS) and the antioxidant defense system in the body [4].

Flavonoids, like antioxidants, have the potential to chelate free radicals by donating hydrogen atoms or unpaired electrons [5]. Antioxidants consumed can prevent various diseases, including flavonoids, which have the potential as inhibitors of the xanthine oxidase enzyme (XOE), thereby inhibiting the formation of xanthine, which then becomes uric acid, and has potential as an inhibitor of acetylcholinesterase (AChE) and tyrosinase [6, 7].

Banana is a plant rich in flavonoids. Among the plants belonging to the genus are the Kepok (*Musa acuminata x balbisiana* Colla, ABB) and Tanduk (*Musa acuminata* Colla, AAB) bananas. Previous studies showed that the ethanol extracts of the peels of both Kepok and Tanduk bananas had antioxidant activity and contained total flavonoids of 0.343 and 0.359 g QE/100 g, and respective total phenolic content of 2.482 and 3.294 g GEA/100 g [8].

Traditionally, plant parts of bananas have been used to treat diseases like fever, cough, bronchitis, dysentery, allergic infections, and diabetes [9]. Banana peel is rich in antioxidant compounds, such as vitamin C, vitamin E, α-carotene, and β-carotene [10]. In addition, several other compounds are also present, including anthocyanins, delphinidins, cyanidins, gallocatechin, catecholamines, β-sitosterol, stigmasterol, campesterol, cycloeucalenol, and cycloartenol [11].

Recently, the development of herbal-based drug delivery systems has continued to develop. The modification of the smaller drug particle size is expected to increase activity. A previous study reported an *in vitro* permeation study of microemulsion preparations containing *Tabernaemontana divaricata* plant extract and its anticholinesterase activity in patients with Alzheimer's disease [12]. The effect of *Schisantherin A* (StA) in nanoemulsion formulation was also reported to significantly increase absolute bioavailability from 4.3% to 47.3%, which was applied as a treatment for Parkinson's disease [13]. Therefore, nanoemulsions provide many advantages in drug delivery systems, especially in treating neurodegenerative diseases that can increase pharmacological activity [14].

Nanoemulsions have many advantages for herbal medicines, including increased solubility rather than increased absorption rate and bioavailability, protection from toxicity, increased pharmacological activity and drug stability, continuous delivery, and protection from physical and chemical degradation [15]. The advantage of other nanoemulsion drug delivery systems is that having a very small droplet size causes an increased reduction in gravitational forces and Brownian motion, which may be sufficient to overcome the creaming or sedimentation phenomenon and prevent droplet flocculation that occurs during storage. Nanoemulsions help dissolve lipophilic drugs and mask the unpleasant taste of some drugs and can be administered from various routes such as topical, oral, and intravenous, providing the possibility of controlled drug release and drug targeting [16, 17].

In this study, an herbal-based drug delivery system was loaded into a nanoemulsion preparation, and this study aimed to test nanoemulsion preparations containing Kepok and Tanduk banana peel extracts (designated as KEP-NE and TAN-NE, respectively) for their antioxidants, as well as acetylcholinesterase and tyrosinase inhibiting activities. Furthermore, the short-term spatial memory improving activity in alloxan-induced mice was also investigated using Y-maze.

## 2 MATERIALS AND METHODS

### 2.1 Plant Materials

Kepok (KEP) and Tanduk (TAN) banana peels were obtained from a traditional market in Bandung-West Java and determined in the Herbarium Bandungense, School of Life Sciences and Technology - Institut Teknologi Bandung. All chemicals and solvents used in this study were of analytical grade.

### 2.2 Extraction of Banana Peel

Reflux extractions were carried out using 96% ethanol as the solvent, in a ratio of 1:3 (crude drug:solvent). The extract obtained was then concentrated using a rotary evaporator.

### 2.3 Preparation of Banana Peel Extract Loaded Nanoemulsion

Nanoemulsion was prepared using a combination of high shear homogenization and ultrasonication [18]. The formula consisted of banana peel extract, oleic acid, Span 80, Cremophor^®^ RH 40, PEG 400, and water. The extract was dispersed in oleic acid, which served as the oil phase. The surfactant Span 80 and Cremophor^®^ RH 40 were added along with the co-surfactant PEG 400. The mixture was stirred at 200 rpm and 70°C. The water phase was introduced gradually into the oil phase at the same temperature. The large-sized particle was pre-milled to improve homogeneity using the high shear homogenization technique within 6 minutes. The particle size was then reduced using a particle size reducing apparatus (Homogeneizador optic Ivymen system CY-500) at an amplitude of 70% for 10 minutes.

### 2.4 Characterization of Nanoemulsions

The characterization of nanoemulsion preparations included the determination of particle size, polydispersity index, and zeta potential using the Delsa™ Nano Zeta Potential and Submicron Particle Size Analyzer (Beckman Coulter). Particle morphology was studied using Transmission Electron Microscope (JEOL JEM 1400).

### 2.5 DPPH Radical Scavenging

The determination of antioxidant activity with DPPH radical reduction was conducted using the Blois method, with minor modifications [8, 19]. The procedure began with preparing a DPPH solution (50 μg/mL) and varying the concentration of the sample solutions. DPPH was mixed with the sample at a ratio of 1:1. The mixture was then incubated at 25°C for 30 minutes in a light-proof test tube. Absorption was measured using a spectrophotometer (Shimadzu UV-1800) at 515 nm. Ascorbic acid was used as the standard control. The percentage of DPPH inhibition was calculated by the formula: [(*A_0_* − *As*) / *A_0_*] × 100%, where *A_0_* is the absorbance of the blank solution, and *As* is the absorbance of the solution containing the extract or standard.

### 2.6 ABTS Radical Scavenging

The determination of antioxidant activity against ABTS radical reduction was conducted using the Re method with minor modifications [20, 21]. Aqueous solutions of 7.6 mM ABTS diammonium salt and 2.5 mM potassium persulfate were prepared. The two solutions were mixed and diluted with pro-analysis ethanol. The final solution was incubated for 12 hours in the dark at room temperature. Absorption was measured using a spectrophotometer (Shimadzu UV-1800) at 734 nm. Ascorbic acid was used as the standard control. The inhibition percentage of ABTS was calculated by the formula: [(*A_0_* − *As*) / *A_0_*] × 100%, where *A_0_* is the absorbance of the blank solution, and as is the absorbance of the solution containing the extract or standard.

### 2.7 Acetylcholinesterase Inhibition Activity

The measurement of acetylcholinesterase inhibitory activity (AChE) was conducted using the colorimetric method, with minor modifications [7]. The reaction was carried out in a 96-well plate. The reagent consisted of 125 μL of 3 mM DTNB in a C buffer (50 mM Tris-HCl, pH 8.0, containing 0.1 M NaCl and 0.02 M MgCl2.6 H2O), 25 μL AChE (0.2 U/mL), 50 μL buffer B (50 mM Tris-HCl pH 8.0 containing 0.1% BSA), and 50 μL sample solutions at different concentrations (50, 100, 200, 400, 600, 800, 1000 µg/mL). The reaction was initiated by adding 25 μL of 15 mM ATCI in water as a substrate. The mixtures in 96-well plates were incubated at 25°C for 15 minutes. The absorbance was measured at 405 nm using a microplate reader (TECAN Infinite 200 Pro). Donepezil HCl was used as the standard. Enzyme inhibition (%) was calculated using the formula: [(*E* − *S*) / *E*] × 100, where *E* is the enzyme activity without the test sample, and *S* is the activity with the test sample.

### 2.8 Tyrosinase Inhibition Activity

The measurement of tyrosinase inhibitory activity was conducted using the colorimetric method, with minor modifications [7]. The reaction mixture was carried out on a 96-well plate. The mixture consisted of 30 μL of tyrosinase solution (1.0 U/mL), 50 μL of phosphate buffer pH 6.8 (0.2 M), 100 μL of sample solutions at different concentrations (200, 400, 600, 800, 1000, 1500 µg / mL), and 50 μL of 10 mM L-DOPA as the substrate. Kojic acid was used as the standard. The mixture was then incubated at 25°C for 15 minutes before measuring the absorbance using a microplate reader (TECAN Infinite 200 Pro) at 475 nm. Enzyme inhibition (%) was calculated using the formula: [(*E* − *S*) / *E*] × 100, where *E* is the enzyme activity without the test sample, and *S* is the activity with the test sample.

### 2.9 Test Animals and Treatment Groups

The test animals were male Swiss Webster mice, weighing 25-35 grams, aged 8-12 weeks. They were kept in a room at 25±3ºC and 70-90% humidity with a 12h:12h light-dark cycle. Mice were induced by alloxan at 120 mg/kg b.w intraperitoneally (i.p.) for three consecutive days to create a memory impairment model [22]. Test substances were given orally for 21 days. Mice were divided into seven groups of five as follows:

Group I: normal saline (normal control)

Group II: alloxan at 120 mg/kg b.w + donepezil HCl at 5.0 mg/kg b.w (positive control).

Group III: alloxan at 120 mg/kg b.w + extract (KEP) at 100 mg/kg b.w.

Group IV: alloxan at 120 mg/kg b.w + extract (KEP) at 300 mg/kg b.w.

Group V: alloxan at 120 mg/kg b.w + NE (KEP-NE) at 100 mg/kg b.w.

Group VI: alloxan at 120 mg/kg b.w + NE (KEP-NE) at 300 mg/kg b.w.

Group VII: alloxan 120 mg/kg b.w + vehicle (negative control).

### 2.10 Short-term Memory Measurement by Y-maze Task

**Fig. (1):**
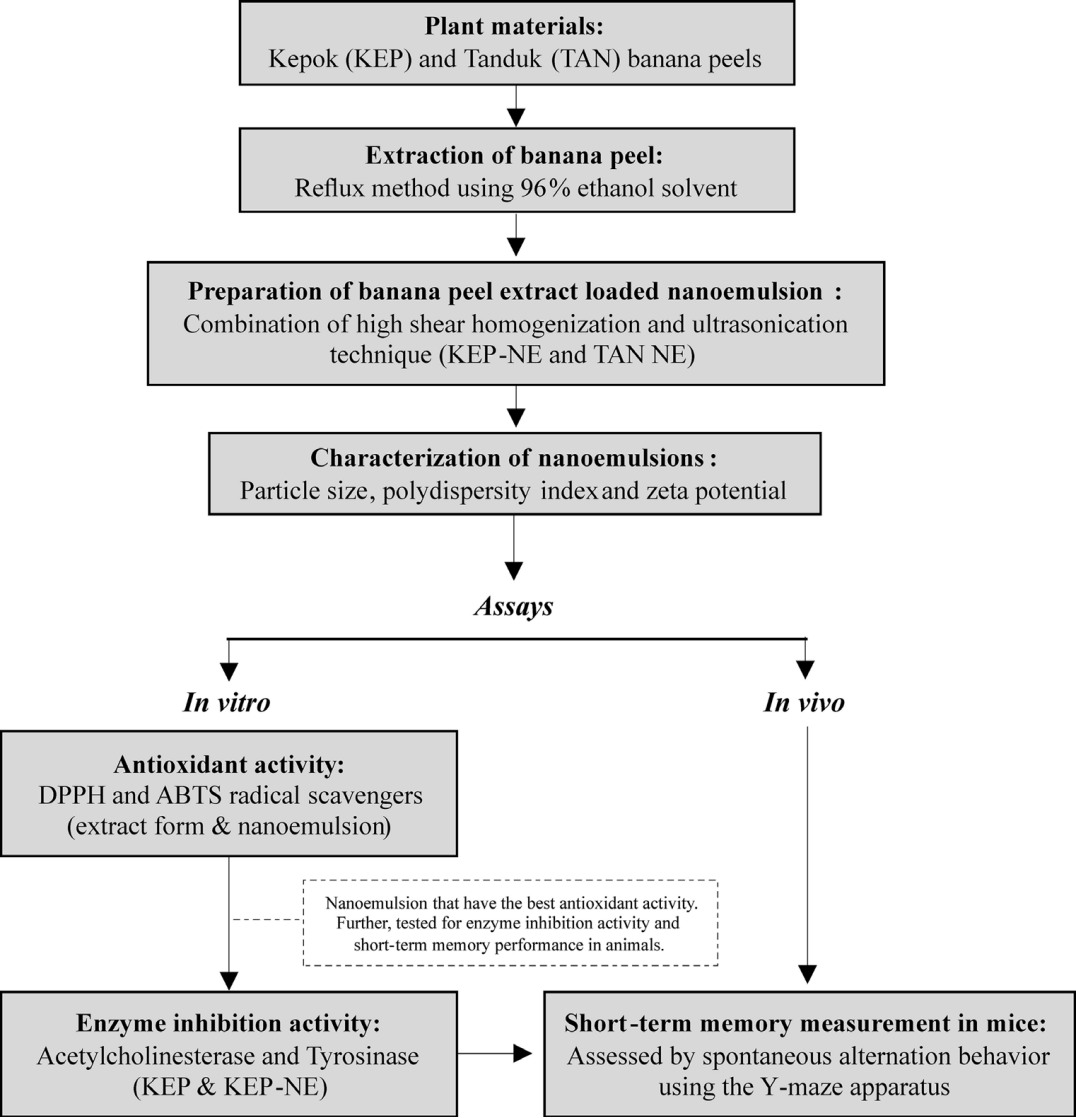
Schematic diagram of the research methodology, starting from the preparation of materials, extraction, and preparation of banana peel extract loaded nanoemulsion to *in vitro* and *in vivo* assays.

Short-term memory measurements were assessed by spontaneous alternation behavior using the Y-maze apparatus [23]. The Y-maze used in this study consisted of three arms (35 cm long, 25 cm high, and 5 cm wide). The behavioral assessment of short-term memory was carried out one hour after administering the test material. The mice were placed in the Y-maze and allowed to explore the maze for 5 minutes. An arm entry was counted when all four legs of the mouse were inside the arm. Spontaneous displacement behavior was defined as including mice in all three arms sequentially and reflecting their spatial working memory. The percentage of spontaneous alternation (%) was calculated using the formula: [Number of Alternations / (Total number of arm entries - 2)] × 100% (Fig. **1**).

### 2.11 Statistical Analysis

Statistical analyses were carried out using Minitab software version 17. Additionally, a one-way ANOVA was used to compare the data among treatment groups, followed by Tukey’s post hoc. A difference was considered significant at *P* < 0.05.

## 3 RESULTS AND DISCUSSION

### 3.1 Characterization of Banana Peel Extract Nanoemulsion Preparation

**Fig. (2):**
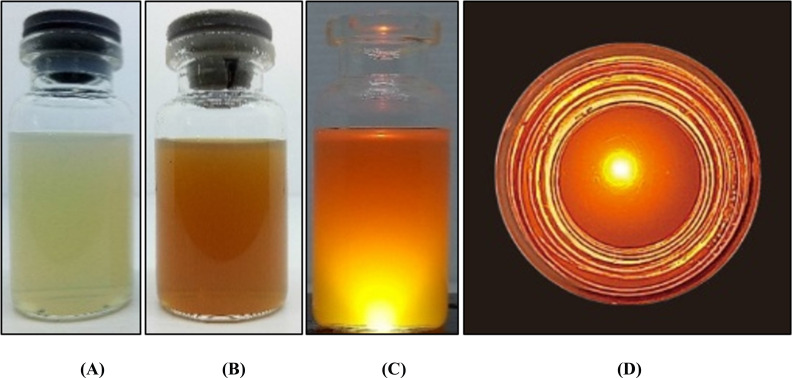
Presentation of NE preparation, (**A**) NE preparation without extract, (**B**) NE preparation containing 1% extract, (**C**) NE preparation illuminated by using LED, and (**D**) Illuminated NE preparation photographed from above.

**Fig. (3):**
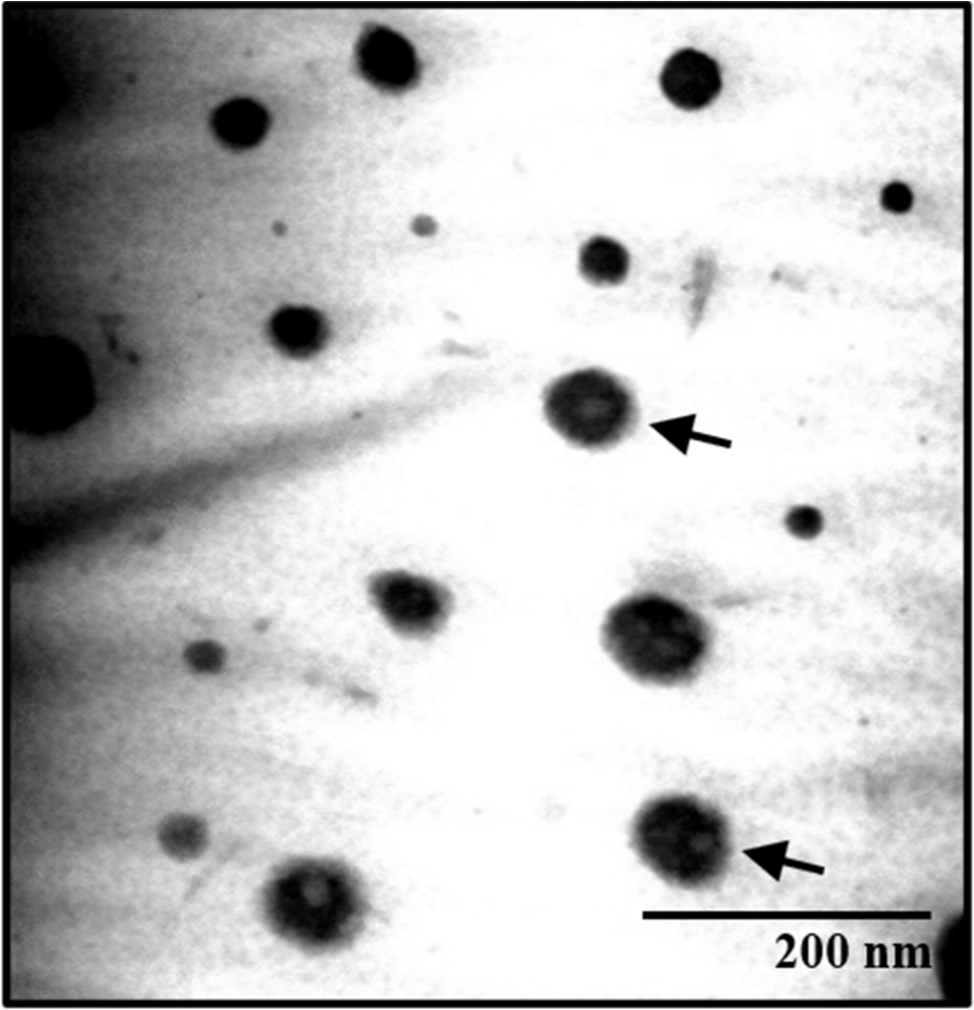
Presentation of globules of Kepok banana peel extracts NE directed by arrow side based on TEM analysis.

The appearance of banana peel extract nanoemulsion was a transparent and brownish liquid (Fig. **2**). Both Tanduk and Kepok banana NE formulations had a particle size of <100 nm (Table **1**). Thus, the preparation met the criteria for nanoparticle preparation, with a particle size between 50-500 nm [24] and polydispersity index values below 0.5, indicating a good particle size distribution and homogeneous particles. The low value of the polydispersity index indicates that the dispersion system formed had a homogeneous particle size distribution to have better stability [25]. The appearance of the developed NE formulation was spherical, as observed based on TEM analysis with a magnification of 20.000x (Fig. **3**). The data on particle size stability of banana peel extract nanoemulsion (KEP-NE and TAN NE) is shown in Fig. (**4**) that the particle sizes of the two preparations are still considered stable (with sizes obtained around 77.83±5,03 to 107.50±6,20) at storage for 21 days at room temperature.

**Fig. (5):**
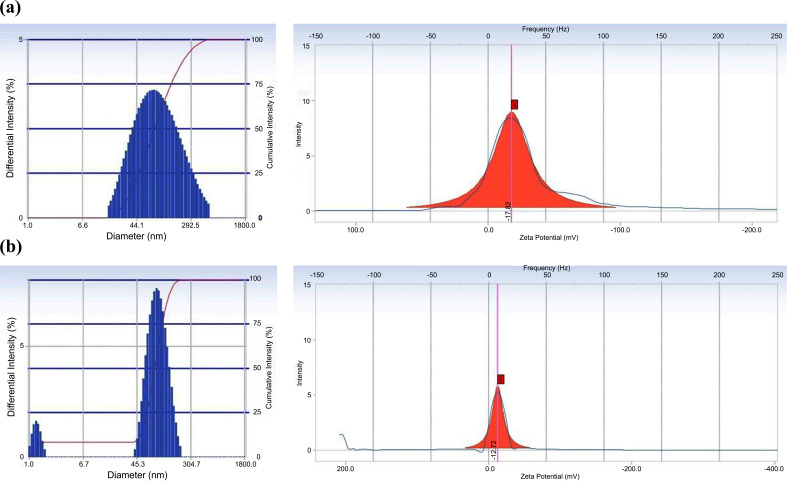
(**a**) Presentation of the mobility distribution of particle size and zeta potential of Kepok banana peel extract nanoemulsion and (**b**) Tanduk banana peel extract nanoemulsion.

The zeta potential values of the two NE preparations were negative. Zeta potential values between -30 mV to +30 mV are generally considered to enable sufficient repulsive force to achieve better colloid physical stability [26]. The zeta potential value is also highly dependent on the composition of the constituents, and the dispersing medium used [27]. Span 80 and Cremophor^®^ RH 40 are nonionic surfactants that can be the components of nanoemulsion and stabilize the emulsion system well. Nonionic surfactants will form steric barriers to maintain the physical stability of the preparation [28]. To illustrate the mobility distribution of particle size and zeta potential of KEP-NE and TAN-NE, see (Fig. **5**).

### 3.2 Determination of Antioxidant Activity by DPPH and ABTS Methods

The determination was based on the value of fifty percent inhibitory concentration (IC_50_), which represents the concentration required for 50% inhibition of free radicals *in vitro*. Antioxidant activities of KEP and TAN are presented in Table **2**.

**Fig. (6):**
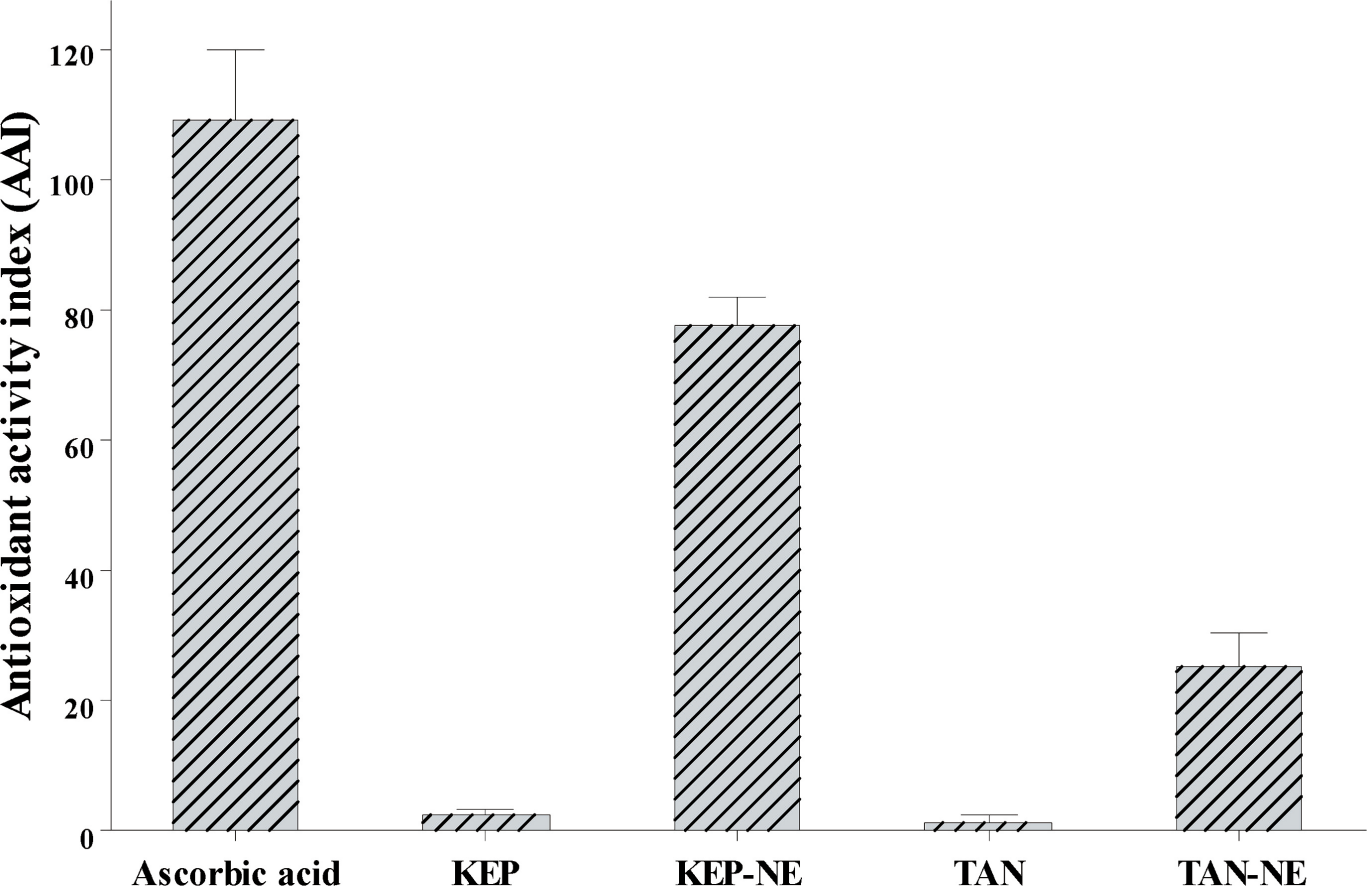
Antioxidant activity of Kepok and Tanduk banana peel extracts and respective NE preparations were tested using the DPPH. The activity is represented as an antioxidant activity index with ascorbic acid as standard.

The free radical scavenging activities by the DPPH method of KEP and TAN were categorized as very strong with IC_50_ of <50 µg/mL, but KEP had a higher activity value than TAN. This result aligns with a previous study [8], demonstrating that Kepok and Tanduk banana peels had IC_50_ values of 6.22 and 101.40 µg/mL, respectively. Both KEP-NE and TAN-NE had IC_50_ values lower than the respective extracts. The excipients of NE had only a very low activity of 3.5% at a 1000 µg/mL concentration. In addition to determining the IC_50_ value, the Antioxidant Activity Index (AAI) was also determined when the DPPH method was used to compare the final DPPH concentration with the IC_50_ value. The AAI values obtained were 109.49±6.42, 2.63±0.44, 77.72±2.45, 1.58±0.52, and 25.40±3.31, respectively (Fig. **6**). The AAI is categorized based on the index, as: weak (<0.5), moderate (0.5-1.0), strong (1.0-2.0), and very strong (>2.0) [29].

The activity of ABTS was higher for KEP than TAN, with respective IC_50_ values of 23.83 and 70.88 µg/mL. For comparison, a study conducted with *Musa acuminata* leaf extract (Simili radjah, ABB) revealed IC_50_ values of 9.0 µg/mL and 187.3 µg/mL for the DPPH and ABTS methods, respectively [30]. While the nanoemulsion dosage form, it provides better ABTS radical scavenging activity than the extract form. This shows that reducing the particle size of the extract can increase the antioxidant activity. It has also been proven by Zorzi *et al.* on the effect of antioxidant activity in nanoemulsion preparations containing extracts of *Achyrocline satureioides* (Lam) [31].

The results further showed that KEP-NE had better antioxidant activity than TAN-NE, using DPPH and ABTS methods. Therefore, KEP was further tested for inhibition of acetylcholinesterase and tyrosinase enzymes and short-term memory performance in animals.

### 3.3 Acetylcholinesterase Inhibition Activity

Acetylcholinesterase is an enzyme involved in the termination of impulse transmission by the rapid hydrolysis of the neurotransmitter acetylcholine in the central and peripheral nervous systems. An acetylcholinesterase inhibitory agent is a molecule that can inhibit the activity of the acetylcholinesterase enzyme, thereby increasing the levels and duration of action of the neurotransmitter acetylcholine [32].

The results of the study of acetylcholinesterase (AChE) inhibitory activity of KEP and KEP-NE are presented in Table **3**. The data showed that KEP-NE was more potent than KEP in inhibiting AChE, which considering the categorization with an IC_50_ value of 108.80 μg/mL, falls into the category of moderate [33]. Taking this finding into consideration, one might suggest that the reduction in particle size contributed to the increased activity, in which the decrease in particle size not only increased surface area but also the higher dissolution of the active ingredients and consequently improved activity. An earlier study further corroborated the AChE-inhibiting activity of pharmaceutical formulations with reduced particle size [34], which found anti-cholinesterase of essential oils from several plants delivered in a microemulsion dosage form. Regarding the active ingredients, the presence of flavonoids might presumably be responsible for the activity. While the exact mechanism has not been fully understood, an early study has shown that AChE inhibition might occur by forming bonds between the aromatic ring of flavonoids containing -OH groups and the anionic active site (Peripheral Anionic Site/PAS) of the enzyme [35].

### 3.4 Tyrosinase Inhibition Activity

Tyrosinase is a key enzyme in the biosynthesis of melanin in skin and hair, but this enzyme has also been expressed at low levels in the human brain and contributes to the formation of neuromelanin. Neuromelanin can biochemically affect neuronal damage associated with Parkinson's Disease [36]. The results of measurements of tyrosinase inhibition activity of KEP and KEP-NE are presented in Table **3**.

The results of the tyrosinase inhibitory activity determination showed that the NE preparation had increased tyrosinase inhibitory activity more than three times the extract. These results could be related to changes in the physicochemical properties of the extract, especially the reduction in particle size and increasing surface area. The benefit of the nanoemulsion drug delivery system was also reported by Roselan *et al*. on the nanoemulsion formulation containing Kojic Monooleate, which showed the highest inhibitory activity (67.12%) as a tyrosinase inhibitor [37].

### 3.5 Short-term Memory Measurement

Changes in acetylcholine levels or AChE enzyme activity can affect the cholinergic transmission process in the brain, causing cognitive deficits and memory disorders [38]. Memory disorders can also be affected by oxidative stress, where free radicals can damage the hippocampal tissue, the brain area essential for memory formation and consolidation.

Alloxan administration produces a redox cycle through the formation of free radicals, leading to damage to several organs such as the eyes, kidneys, and brain. The gradual progressive changes in the brain have been related to poor memory performance. As also explained, oxidative stress is a major risk factor for cognitive failure in alloxan-induced mice [22].

Spontaneous alternation behavior in the Y-maze test has been seen as an indicator of short-term spatial memory [23]. A high percentage of spontaneous alteration represents good working memory [39]. The results of the measurement of the spontaneous alternation behavior are presented in Fig. (**7**). In alloxan-induced mice, KEP and KEP-NE at respective doses of 100 and 300 mg/kg significantly improved their memory performance. However, there were no significant differences among these treatments.

The total numbers of entries in mice treated with KEP and KEP NE at 300 mg/kg were not significantly different than those treated with KEP at 100 mg/kg, albeit differences in the % value of spontaneous change. This was consistent with the results of previous studies demonstrating that the change in % of spontaneous alternation was not only related to the total number of entries but was also influenced by an increase in the number of spontaneous behaviors [40].

The increase in locomotor activity as assessed by the total number of entries in each arm of the Y-maze and the behavior of spontaneous replacement was shown to increase the percentage of spontaneous alternation. A close observation of the results revealed that the activity was not dose-dependent. The highest activity was shown in the group that received KEP-NE at 100 mg/kg. Less activity in higher doses might be related to higher levels of components with opposite effects [41, 42].

This study showed that the drug delivery system in nanoemulsion preparations affected therapeutic activity in treating alloxan-induced memory disorders as measured by spontaneous alternation behaviors. Another study conducted by Chen *et al.* [43] has proven that the nanoemulsion preparation of black garlic extract has a better effect than the extract form on lowering blood pressure and improving learning/memory abilities in rats.

The nanoemulsion drug delivery system also benefits curcumin, one of the most widely used bioactive compounds in traditional medicine, including antioxidant, anticancer, antihypertensive, antidiabetic, and antineuroinflammatory effects (due to memory loss). However, due to low solubility and lower bioavailability, the development of nanoemulsion technology containing curcumin can overcome the weakness of these compounds [44].

## CONCLUSION

Kepok banana peel extract (KEP) and Tanduk banana peel extract (TAN) could be formulated into nanoemulsion (NE) dosage forms, which had improved antioxidant and AChE as well as tyrosinase inhibiting activity compared to the extracts. In addition, using the spontaneous alteration paradigm, both extracts and the nanoemulsion formulation ameliorated spatial memory in alloxan-induced mice. This study shows that extracts and nanoemulsions can be alternative treatments for memory disorders. It is revealed that the modification of the smaller drug particle size makes an active contribution to the delivery system, and extract-loaded nanoemulsion could increase pharmacological activity.

## Figures and Tables

**Fig. (4):**
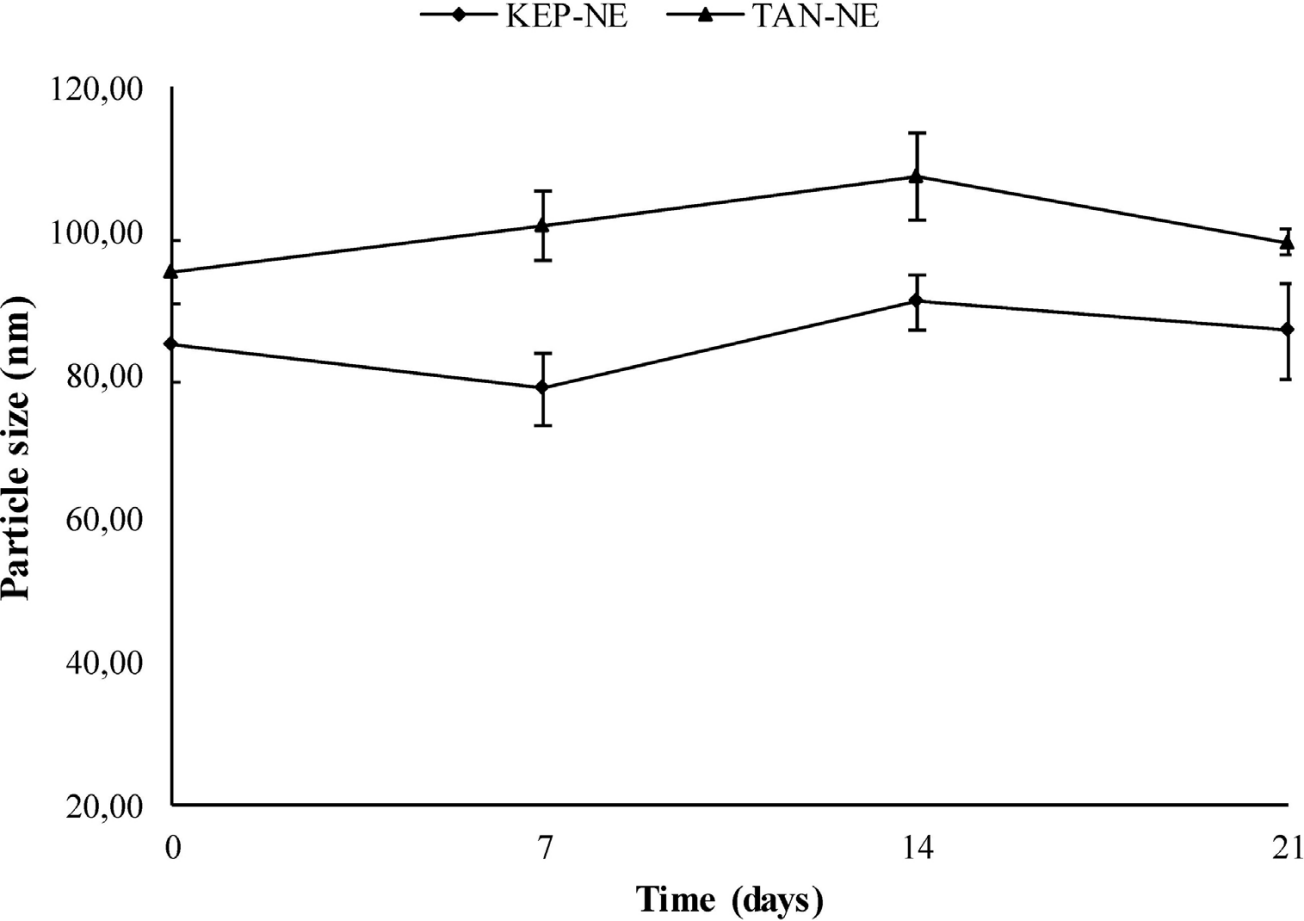
Particle size stability of banana peel extracts nanoemulsion preparations (KEP-NE and TAN NE) storage for 21 days at room temperature.

**Fig. (7):**
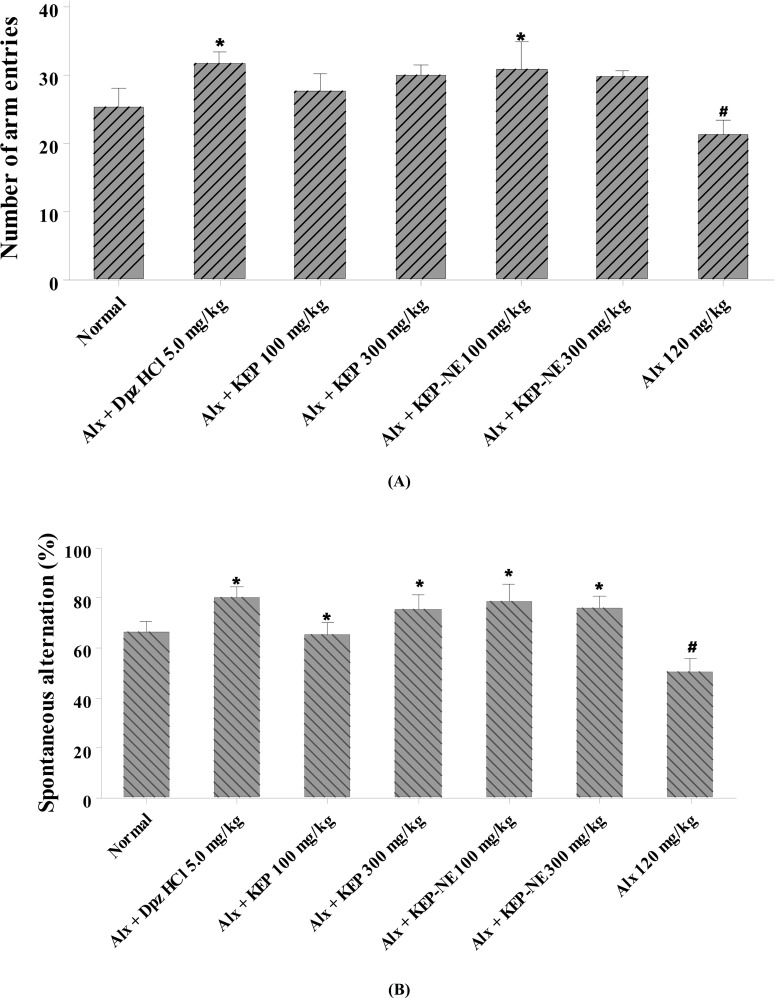
(**A**) Effects of KEP and KEP-NE on spatial memory in alloxan-induced mice, represented by the number of arm entries (**B**) and % spontaneous alterations upon testing in the Y maze. The test substance was given for 21 days immediately after the effect of alloxan (120 mg/kg, i.p.) was confirmed. Alx=alloxan, Dpz=donepezil HCl, KEP=Kepok banana peel extract, KEP-NE=nanoemulsion preparation of Kepok banana peel extract. The data represent the mean ± SD of 5 mice. ^#^*P* < 0.05 vs. normal group, *P < 0.05 *vs*. Alx group, using a one-way ANOVA followed by Tukey’s post hoc test.

**Table 1:** Physical characteristics of banana peel extract nanoemulsions.

**Test Substance**	**Particle Size (nm)**	**Polydispersity Index (PI)**	**Zeta Potential (mV)**
KEP-NE	84.2 ± 5.4	0.280 ± 0.06	-22.63 ± 1.80
TAN-NE	94.1 ± 4.4	0.282 ± 0.03	-16.97 ± 1.14

**Note:** Data represent mean ± SD, *n* = 3.

**Table 2:** Antioxidant effects of KEP and TAN, and the respective NE, were assessed using DPPH and ABTS methods.

**Test substance**	**DPPH (µg/mL)**	**ABTS (µg/mL)**
KEP	18.98 ± 2.87^a^	23.87 ± 1.96^a^
TAN	31.69 ± 1.85^b^	70.88 ± 1.26^b^
KEP-NE	0.64 ± 0.50^c^	1.10 ± 1.02^c^
TAN-NE	1.97 ± 1.18^c^	1.72 ± 0.18^c^
Ascorbic acid	0.46 ± 0.04	0.52 ± 0.02

**Note:** Data represent mean ± SD, *n* = 3. Means with the same alphabet along the column are not significantly different (*P* < 0.05) using a one-way ANOVA.

**Table 3:** Acetylcholinesterase & tyrosinase inhibitory activities of Kepok banana extract and its nanoemulsion preparation.

**Test Substance**	**Acetylcholinesterase (µg/mL)**	**Tyrosinase (µg/mL)**
KEP	389.72 ± 11.81^a^	834.30 ± 2.68^a^
KEP-NE	108.80 ± 8.19^b^	251.47 ± 8.54^b^
Donepezil HCl	10.35 ± 0.22^c^	-
Kojic acid	-	1.93 ± 0.10^c^

**Note:** Data represent mean ± SD, *n* = 3. The different letters indicate significant differences between samples (*P* < 0.05) using a one-way ANOVA.

## Data Availability

The data that support the findings of the study is available upon request from the corresponding author [RM].

## LIST OF ABBREVIATIONS

ROS: Reactive Oxygen Species
XOE: Xanthine Oxidase Enzyme
AChE: Acetylcholinesterase
KEP: Kepok
TAN: Tanduk
